# Optimising a 3D convolutional neural network for head and neck computed tomography segmentation with limited training data

**DOI:** 10.1016/j.phro.2022.04.003

**Published:** 2022-04-28

**Authors:** Edward G.A. Henderson, Eliana M. Vasquez Osorio, Marcel van Herk, Andrew F. Green

**Affiliations:** a The University of Manchester, Oxford Rd, Manchester M13 9PL, UK; b Radiotherapy Related Research, The Christie NHS Foundation Trust, Manchester M20 4BX, UK

**Keywords:** Limited data, 3D convolutional neural network, CT scan auto-segmentation

## Abstract

**Background and purpose:**

Convolutional neural networks (CNNs) are increasingly used to automate segmentation for radiotherapy planning, where accurate segmentation of organs-at-risk (OARs) is crucial. Training CNNs often requires large amounts of data. However, large, high quality datasets are scarce. The aim of this study was to develop a CNN capable of accurate head and neck (HN) 3D auto-segmentation of planning CT scans using a small training dataset (34 CTs).

**Materials and Method:**

Elements of our custom CNN architecture were varied to optimise segmentation performance. We tested and evaluated the impact of: using multiple contrast channels for the CT scan input at specific soft tissue and bony anatomy windows, resize vs. transpose convolutions, and loss functions based on overlap metrics and cross-entropy in different combinations. Model segmentation performance was compared with the inter-observer deviation of two doctors’ gold standard segmentations using the 95th percentile Hausdorff distance and mean distance-to-agreement (mDTA). The best performing configuration was further validated on a popular public dataset to compare with state-of-the-art (SOTA) auto-segmentation methods.

**Results:**

Our best performing CNN configuration was competitive with current SOTA methods when evaluated on the public dataset with mDTA of (0.81±0.31) mm for the brainstem, (0.20±0.08) mm for the mandible, (0.77±0.14) mm for the left parotid and (0.81±0.28) mm for the right parotid.

**Conclusions:**

Through careful tuning and customisation we trained a 3D CNN with a small dataset to produce segmentations of HN OARs with an accuracy that is comparable with inter-clinician deviations. Our proposed model performed competitively with current SOTA methods.

## Highlights

• Convolutional neural networks (CNNs) are used for auto-segmentation in radiotherapy.
• However, CNNs rely on large, high-quality datasets: a scarcity in radiotherapy.
• We develop a CNN model, trained with limited data, for accurate segmentation.
• Multiple experiments were performed to optimise key features of our custom model.
• Our model is competitive with state-of-the-art methods on a public dataset.

## 1 Introduction

The 3D segmentation of organs-at-risk (OARs) is a crucial step in the radiotherapy pathway. However, segmentation or delineation by clinicians is slow, expensive and prone to inter- and intra-observer variability even among experienced radiation oncologists [1]. Fully convolutional neural networks (CNNs) are now the state-of-the-art for automated medical image segmentation [2]. Recently, a considerable number of methods are have been proposed and implemented to perform segmentation faster and with higher consistency [3], [4], [5], [6], [7]. Cutting-edge radiotherapy workflows use auto-segmentation models to suggest contours which experienced radiographers will confirm and edit if required [8].

Supervised training of CNN models traditionally requires large amounts of high quality annotated data (often *>*1000s of examples) [9]. In this application full volumetric segmentation by radiographers, ideally with the same level of expertise and following the same guidelines, is needed for every image. As a result, high-quality sets of training data for auto-segmentation are often limited in size. Large institutions and commercial systems regularly use datasets containing 100s of images [10], [11]. However, very few researchers have access to such large datasets. For 2D tasks, transfer learning from large, pre-trained backbone models, such as ResNet, is often used to improve performance when limited training data is available. Analogous backbone models are not yet readily accessible in 3D.

This study aimed to develop a custom 3D CNN model capable of accurate auto-segmentation of head and neck (HN) OARs using a small, publicly available dataset (34 CTs) for training. The design space of CNN models is extensive and in addition to the volume of training data available, choices in the CNN architecture and training protocols can heavily impact model performance. We selected three key design elements to optimise in the development of our custom CNN.

## 2 Materials and methods

### 2.1 CNN model architecture

**Fig. 1:**
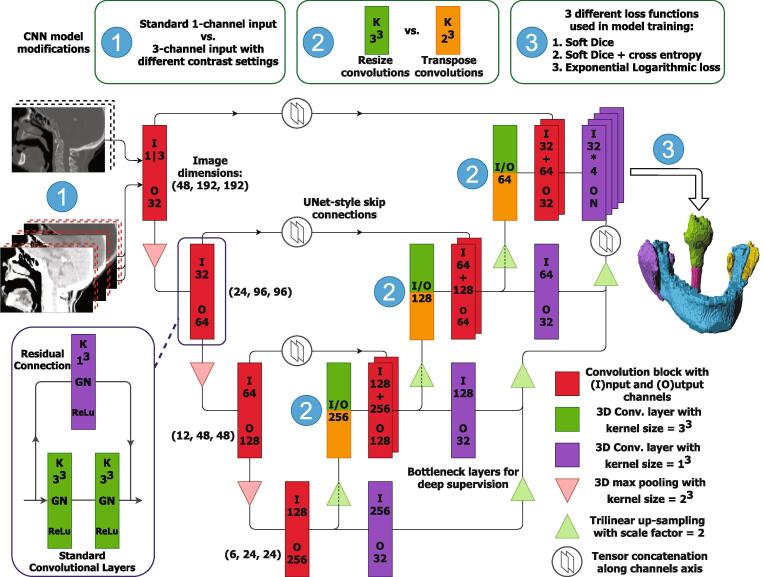
The CNN architecture used in this study. The base model used was a 3D Res-UNet with deep supervision. In this figure we highlight the three modifications that form the presented experiments. We compared using multiple contrast settings for the model input (1), resize or transpose convolutions in the decoder portion (2) and three different loss functions (3). When using transpose convolutions (orange), we did not perform tri-linear up-sampling.

Our base segmentation CNN was founded on the 3D UNet design [12]. This consists of an encoding pathway of repeating zero-padded 3x3x3 convolutional and pooling layers, followed by a decoding pathway of similar convolutions and up-sampling (Fig. 1). Residual skip connections were added to smooth the training process. These residual connections were implemented with 1x1x1 convolutional layers to match the channel number on either side of the convolutional block [13]. Multi-level deep supervision was introduced at each level in the decoding portion of the network to accelerate convergence. The deep supervision connections contain bottleneck 1x1x1 convolutions reducing the number of model parameters and enabling training on a single graphics processing unit (GPU).

### 2.2 Multiple input channels with specific contrast settings

Generally, images are pre-processed before being used as input for a CNN. Routine pre-processing consists of normalising images to have μ=0 and σ=1 or mapping the image onto the range [0,1].

An advantage of working with computed tomography (CT) scans is that voxel intensities are calibrated to Hounsfield units (HU), a scale of tissue density with fixed reference values at air (-1024 HU) and water (0 HU). Clinicians use windowing or grey-level mapping when visualising CT images to enhance the contrast of different tissues and highlight particular structures, for example, narrow windows are used for soft tissues with similar attenuation and wide windows for visualising bone. Image brightness is adjusted with the window level (*L*) and contrast is adjusted with the window width (*W*).

**Fig. 2:**
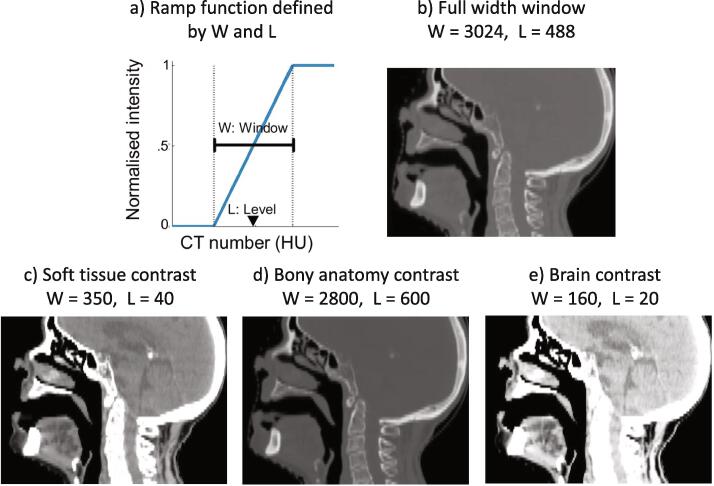
a) The windowing ramp function to map CT image intensities. b) Contrast settings for the full-width window baseline approach. c-e) Window width and level contrast settings selected for our multiple input channels approach.

*L* and *W* define a ramp function that is used to map all intensities in a given image as shown in Fig. 2. Our proposed approach used three input channels, normalised with distinct contrast settings. The chosen *W* and *L* contrast settings are used by radiologists to specifically view soft tissue, bony anatomy and brain tissue [14]. The three distinctly contrasted CT volumes were concatenated along the “channels” axis and fed through the CNN simultaneously. This approach is analogous to separate RGB channels in 2D natural images.

We compared our proposed method with a comparison baseline that used a single input channel where the CT image is normalised with a full-width window. Practically this involved setting L=488 and W=3024, standardising the entire intensity range of the image onto the range [0,1].

### 2.3 Resize convolutions

Transpose convolutions are frequently used to mimic the inverse convolution operation, increasing an image’s spatial dimensions by dilating the input [15]. However, transpose convolutions can produce checkerboard artefacts in CNN-generated images [16]. Resize convolutions (up-sampling followed by a standard convolutional layer) have been proposed as a drop-in replacement to remedy such artefacts. We compare model configurations using traditional transpose and resize convolutions in the decoder portion of the segmentation CNN. In this work, 3D resize convolutions were implemented using tri-linear up-sampling prior to a zero-padded 3x3x3 convolutional layer.

The increase in CNN model size was negligible when using multiple input channels (4,896,693→4,897,621 parameters). Resize convolutions use 3x3x3 kernels, which added many more parameters when compared to transpose convolutions which use 2x2x2 kernels (4,896,693→6,530,997 parameters). Model configurations using three input channels and resize convolutions contained 6,531,925 parameters.

### 2.4 Loss functions

We performed experiments with three loss functions: a simple overlap metric; a linear combination of an overlap metric and cross-entropy; and a similar combination with added non-linearities.

In segmentation tasks, overlap loss metrics have gained popularity due to their easy implementation and quick convergence. The first metric we evaluated was the multi-class weighted Soft Dice (wSD) loss function which is based on the Dice similarity coefficient (DSC). The wSD loss is given by

(1) $$L_{\mathrm{wSD}}=w_{l}\left(1-\frac{2\underset{V}{\sum }y_{\mathrm{true}}\cdot y_{\mathrm{pred}}+1}{\underset{V}{\sum }y_{\mathrm{true}}+\underset{V}{\sum }y_{\mathrm{pred}}+1}\right)$$

where *V* is the CT volume and *l* is the OAR label. In 3D volumes there is often a significant class imbalance between background and labelled voxels which can be several orders of magnitude, especially for small OARs. OAR-specific weights, *w*_*l*_, are often added to address class-imbalance. In this study, weights for each OAR were calculated with the inverse label frequency as

(2) $$w_{l}=\frac{{\left(\frac{\underset{l}{\sum }y_{l}}{\underset{V,l}{\sum }y_{l}}\right)}^{\alpha }}{\underset{l}{\sum }{\left(\frac{\underset{l}{\sum }y_{l}}{\underset{V,l}{\sum }y_{l}}\right)}^{\alpha }},$$

with α=1/3.

Cross-entropy (XE) is a popular loss function that evaluates target and prediction similarity with log-probabilities. The second metric implemented was composed of a linear combination of wSD and weighted XE (wSD + XE).

Wong et al. proposed an “Exponential Logarithmic Loss” function (ExpLogLoss) for segmentation of objects with high unbalanced object sizes. This loss function was originally designed for segmenting 3D brain MR images and is formed of a sum of logarithmic SD and weighted XE. We evaluated the impact of using an ExpLogLoss function with the suggested settings outlined in [17]. The ExpLogLoss function was calculated as

(3) $$L_{\mathrm{ELL}}=E[(-\ln{\left({\mathrm{Dice}}_{l}\right)}^{0.3}]+E[w_{l}{\left(-\ln(p_{l}(x))\right)}^{0.3}]$$

where *Dice*_*l*_ was the Dice similarity coefficient for OAR *l*, -ln(*p*_*l*_(**x**)) was the negative log likelihood loss and α=0.5 for *w*_*l*_.

### 2.5 Implementation details

All our models were implemented in PyTorch 1.6.0. All network training was performed on a 16 GB NVidia Tesla V100 GPU. Individual model training took ∼5hrs. Segmentation inference took <1s per 3D CT image.

Extensive data augmentation was used to improve the robustness of the model. This was essential to prevent over-fitting when using a small training dataset. Throughout training the original CT images and gold standard segmentation masks were transformed with random sequences of augmentations. The 3D augmentation operations include: lateral mirroring (with probability, p=0.5); shifting of ±4 voxels maximum in each direction (p=1,±4 mm in-plane & ±10 mm axially); rotations between ±10° to imitate cervical flexion, extension and rotation (p=0.75); and volumetric scaling between 90–110% (p=0.5). All augmentations were implemented using the *numpy* and *scipy* libraries.

The Adam optimiser was used with an initial learning rate of 10^−2^, which was reduced by a factor of 10 each time the validation loss plateaued for 100 epochs. Models were allowed to train for up to 1000 epochs, with early stopping implemented if the validation loss failed to improve for 250 epochs. Due to the size of the 3D CNN and input CT volumes, the batch size was restricted to one. However, gradient accumulation was used to delay model parameter updates, which simulated a batch size of four.

### 2.6 Data and experimental setups

For model development we used a publicly-available open dataset of 34 CT images (https://github.com/deepmind/tcia-ct-scan-dataset) [11]. Each of the 34 HN CTs, with voxel resolution of 1x1x2.5 mm, have OAR delineations from two doctors. One set of delineations was treated as the gold standard and used for training. The CNN model was trained for 3D segmentation of the mandible, brainstem, parotid glands and the cervical section of the spinal cord. We performed experiments to assess every configuration of the three loss functions, multiple- vs. single-channel contrast input and resize vs. transpose convolutions. Before segmentation the CTs were automatically cropped to anatomically consistent sub-volumes with the dimensions of 200x200x56 voxels using in–house software [18].

A 5-fold cross-validation was performed for each model configuration [19]. In each fold, a CNN model was trained from scratch using 24 training images and 3 validation images. In such a cross-validation, training data is used to adjust model parameters, whereas the validation data informs adjustments to the learning rate and when to terminate the training process. Sets of 7 testing images were held out and used to evaluate the final segmentation performance of the fold.

### 2.7 Segmentation performance metrics

Model segmentation performance was compared to the measured deviation between the two doctors, using both the 95th percentile Hausdorff distance (HD95) and mean distance-to-agreement (mDTA). A Wilcoxon signed-rank test of the second clinician and CNN HD95 samples was performed for each OAR with the null hypothesis that the differences of the medians are zero.

Overlap metrics such as DSC and the Jaccard index are often reported for semantic segmentations works. However, such metrics are heavily biased towards structure volume, insensitive to fine details as bulk overlap can hide clinically relevant differences between structure boundaries [3], [20]. In radiotherapy, small deviations in the borders of segmentations can have a potentially serious impact, e.g. increasing the risk of side effects for the patient through unplanned irradiation of an OAR.

As such, distance metrics, such as the mDTA and HD95 [21], are preferred [22] and reported in this paper. To calculate these metrics, distance transform maps were created for the reference segmentation and sampled on the voxels on the boundary of the evaluated segmentations. We evaluated these distances symmetrically, i.e. using distance maps from the golden standard and sampling on the boundary voxels of the predicted segmentation and vice versa. These distances were then summarised by their mean (mDTA) and by their 95th percentile maximum distance (HD95). mDTA serves to assess the overall results and HD95 the worst matching region.

### 2.8 External validation

Our optimal model configuration was further validated on the public MICCAI Head and Neck Auto Segmentation Challenge 2015 dataset (version 1.4.1) [23]. This dataset (MICCAI’15 set) contains 48 patients which were originally divided into; 25 for training, 8 for optional additional training, 10 for offsite testing and 5 for onsite testing. We retrained our best configured model using the original set of 25 for training, the 5 onsite testing images for validation and the 10 offsite testing images for testing. The 8 samples in the original “optional training” set do not have all OARs delineated so were not included. Unfortunately, this dataset does not contain spinal cord delineations.

Our proposed model’s results on the MICCAI’15 set were compared to the state-of-the-art (SOTA) results published on the same dataset by Huang et al. [4], Zhang et al. [5], Gao et al. [6], Gou et al. [7] and Kawahara et al. [27]. Each comparison method published either the HD95, the average surface distance (ASD), equivalent to mDTA, or neither of these. However, all five studies published DSC results, so we additionally calculated DSC results for our model on the MICCAI’15 set for comparison.

## 3 Results

### 3.1 Model development

Descriptive results of the HD95 and mDTA metrics for every model configuration are shown in Table 1.

**Table 1:** Median values of the HD95 and meanDTA metrics for every model configuration. Lower values show closer agreement between the CNN predicted segmentations and the gold standard. In this table *T* and *R* indicate models using either Transpose or Resize convolutions in the decoder portion respectively. These results are summarised by the median and standard deviation of the metrics for each OAR across all patients in the five test set folds. The best performing configurations for each OAR are highlighted in bold font and are determined with more significant figures than shown. For the HD95, the majority of the spinal cord results reflect the CT image slice thickness (2.50 mm). This suggests most models made errors in the spinal cord length by a single slice. Model configurations trained with the ExpLogLoss function consistently produce better segmentations.

Loss	Conv.	In-ch.	Brainstem	Mandible	L Parotid	R Parotid	Spinal cord
*HD95* (mm)							
wSD	T	1	4.5±1.4	1.1±0.8	5.1±2.8	5.2±2.5	2.5±1.5
	T	3	4.1±1.4	1.1±0.9	4.1±3.9	4.9±3.4	2.5±1.3
	R	1	4.6±1.2	1.1±0.8	6.1±4.0	5.7±2.5	2.6±2.4
	R	3	4.0±1.9	1.2±0.7	5.0±4.2	5.3±3.6	2.5±1.3
wSD + XE	T	1	4.4±1.4	1.0±0.4	5.8±2.9	5.9±3.1	2.5±1.4
	T	3	3.9±1.4	1.2±0.7	4.7±3.4	5.0±3.3	2.7±1.5
	R	1	4.9±1.6	1.1±0.6	5.9±2.4	5.5±2.7	2.5±1.7
	R	3	3.5±1.4	1.3±0.7	4.6±3.2	4.8±3.5	2.5±1.7
Exp Log Loss	T	1	4.1±1.3	1.0±0.4	5.0±2.2	5.8±2.9	2.5±1.5
	T	3	3.4±1.5	1.0±0.6	4.4±3.4	4.8±2.8	2.5±1.4
	R	1	4.1±1.7	1.0±0.6	4.9±3.4	5.1±3.2	2.5±1.3
	R	3	3.4±1.7	1.2±0.8	4.0±3.6	4.6±2.6	2.5±3.6
Doctor comparison			2.5±0.8	1.0±0.5	3.9±4.9	3.9±2.4	2.0±3.6
							
*mDTA* (mm)							
wSD	T	1	1.1±0.4	0.2±0.1	1.1±0.4	1.2±0.5	0.5±0.3
	T	3	1.1±0.3	0.2±0.1	1.0±0.5	1.1±0.6	0.5±0.2
	R	1	1.3±0.4	0.2±0.1	1.3±0.5	1.3±0.5	0.6±0.3
	R	3	1.1±0.4	0.2±0.1	1.2±0.5	1.3±0.6	0.5±0.2
wSD + XE	T	1	1.5±0.4	0.1±0.1	1.4±0.6	1.5±0.6	0.6±0.2
	T	3	1.1±0.4	0.2±0.1	1.1±0.4	1.1±0.6	0.5±0.3
	R	1	1.5±0.4	0.2±0.1	1.5±0.6	1.4±0.6	0.6±0.3
	R	3	1.1±0.4	0.2±0.1	1.1±0.5	1.1±0.7	0.5±0.2
Exp Log Loss	T	1	1.2±0.4	0.1±0.1	1.1±0.4	1.2±0.6	0.5±0.2
	T	3	0.9±0.4	0.1±0.1	1.0±0.5	0.9±0.5	0.5±0.2
	R	1	1.2±0.4	0.1±0.1	1.2±0.6	1.2±0.7	0.6±0.2
	R	3	1.0±0.5	0.1±0.1	0.9±0.4	1.0±0.5	0.5±0.6
Doctor comparison			0.6±0.2	0.1±0.1	0.8±0.4	0.8±0.3	0.4±0.2

All models had similar HD95 performance on the spinal cord with the results consistently reflecting the CT slice thickness (2.5 mm). This suggests most models made errors in the spinal cord length by a single slice. Otherwise, across all OARs and both metrics, our consistently top-performing models were trained with the ExpLogLoss function.

**Fig. 3:**
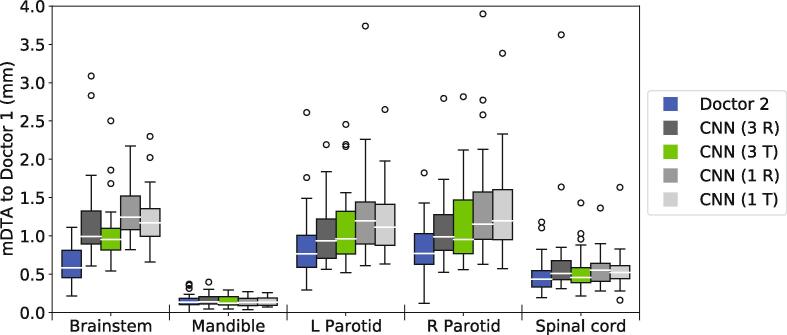
Boxplots comparing the mDTA for the four model configurations trained using the best performing loss function, ExpLogLoss, and the deviation between doctors for reference (blue boxes). For this figure, lower values indicate better segmentations. Configurations using 3-channel input (3 R&T) outperform the single-channel counterparts (1 R&T) in all soft tissue OARs. Models with traditional transpose convolutions (T) produce marginally better segmentations, with the best-performing model highlighted in green.

To more closely examine models trained with the ExpLogLoss function, we compared the mDTA values for all such model configurations using box-plots in Fig. 3. The best performing model configuration used multiple input channels, transpose convolutions and was trained using the ExpLogLoss function. This configuration produced parotid gland, spinal cord and mandible segmentations with a similar level of accuracy to inter-clinician deviation. The only significant difference was found for the brainstem (p=0.00008, Wilcoxon signed-rank test). However, the segmentation performance in the brainstem was still good, with a median HD95 of (3.37±1.50)mm and median mDTA of (0.95±0.37) mm.

Model configurations with multiple input contrast channels consistently outperformed the single input channel counterparts for segmentation performance in the soft tissue organs (brainstem, parotid glands and spinal cord). The segmentation performance was equivalent in the mandible regardless of input type.

When training with wSD and the wSD + XE combination loss functions, the transpose and resize convolutions performed very similarly. However, when training with the ExpLogLoss function, the transpose convolutions performed marginally better.

### 3.2 External validation

Our best-performing model configuration (three-channel input, transpose convolutions and ExpLogLoss function) was then re-trained and evaluated on the MICCAI’15 set. In Table 2 the HD95, mDTA and DSC metric results for our proposed method are presented.

**Table 2:** HD95, ASD/mDTA and DSC comparison results on the MICCAI’15 set. Bold font indicates the best performing model. Dashes indicate that results for the OAR are not reported. *Kawahara et al. reported a single DSC for the parotids.

*OAR*	Brainstem	Mandible	Left Parotid	Right Parotid
*HD95* (mm)				
Gao et al. [6]	**2.32** ± **0.70**	1.08 ± 0.45	**1.81** ± **0.43**	**2.43** ± **2.00**
Gou et al. [7]	2.98 ± 0.61	1.40 ± 0.02	3.48 ± 1.28	3.15 ± 0.67
Ours	2.83 ± 1.05	**1.00** ± **0.73**	2.87 ± 0.89	3.55 ± 1.35
*ASD/ mDTA* (mm)				
Huang et al. [4]	1.28 ± 0.45	0.56 ± 0.27	0.86 ± 0.24	1.02 ± 0.38
Gou et al. [7]	1.19 ± 0.16	0.47 ± 0.11	1.21 ± 0.34	1.14 ± 0.22
Ours	**0.81** ± **0.31**	**0.20** ± **0.08**	**0.77** ± **0.14**	**0.81** ± **0.28**
*DSC*				
Huang et al. [4]	87.9 ± 2.4	91.6 ± 2.1	88.4 ± 1.5	87.8 ± 2.0
Zhang et al. [5]	**91** ± **2**	**95** ± **3**	87 ± 3	87 ± 7
Gao et al. [6]	88.2 ± 2.5	94.7 ± 1.1	**89.8** ± **1.6**	**88.1** ± **4.2**
Gou et al. [7]	88 ± 2	94 ± 1	87 ± 3	86 ± 5
Kawahara et al. [27]	88	-	81*	81*
Ours	88.3 ± 3.6	93.4 ± 1.9	88.6 ± 1.6	87.2 ± 3.1

**Fig. 4:**
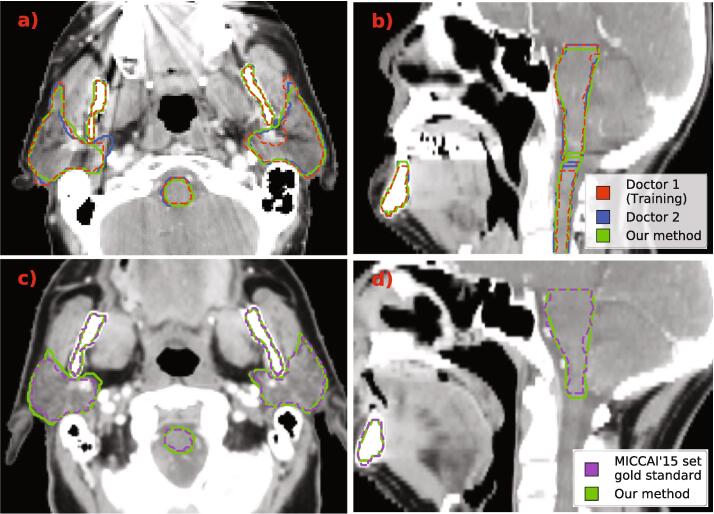
Example segmentations produced by our CNN model (green). In the top row, a) and b), we show 2D axial and sagittal views of a patient from the dataset we used for model development. This dataset contained segmentations produced by two doctors which are shown and red and blue. On the bottom row, c) and d), we show axial and sagittal 2D slices of a patient from the MICCAI’15 set. The gold-standard segmentations for this set are shown in purple.

In Fig. 4 we illustrate example segmentations produced by our proposed method. Fig. 4, Fig. 4b show 2D axial and sagittal slices of a patient from our original dataset. Fig. 4, Fig. 4d are examples of a patient from the MICCAI’15 set used for external validation.

## 4 Discussion

CNNs, which are now being used for auto-segmentation in radiotherapy planning, typically require large datasets to train effectively. We developed a CNN model capable of accurate HN CT segmentation when trained on a small dataset. This was achieved through careful tuning of a customised 3D CNN. Varying particular elements of our model provided insight into what impacts the performance of CNN auto-segmentation methods, in particular UNet-based architectures.

Using multiple contrast settings for the model input was a key strategy to improve segmentation performance for soft-tissue OARs. We compared our three pre-selected contrast channels to a baseline input using a single full-width window. We did not exhaustively compare each contrast window individually or in combinations as this would have greatly increased the number of experiments. It is possible that using just one of our contrasted channels could be sufficient in some situations (e.g. for the mandible). However, identifying these specific situations adds considerable complexity to the auto-segmentation task and using all three channels adds little computational load in both training and inference stages. Additionally, we did not optimise the contrast settings used in this study, instead relying on values sourced from literature [14]. In 2018, Lee et al. developed a window setting optimisation module that implements contrast normalisation as a learnable parameter of the model [24]. It would be interesting to discover whether a similar module could be deployed successfully within our methodology.

The loss function is a crucial component of training a deep learning model. We found the ExpLogLoss function, originally developed by Wong et al. [17], produced higher accuracy segmentation models compared to simpler soft Dice and cross-entropy combination functions. Lu et al. reported concurring results for a similar loss function when applied to 3D stroke lesion segmentation in T1 weighted MR images [25]. In future work it would be of interest to evaluate the recently introduced “Unified Focal” loss function which performs well for highly imbalanced class segmentation [26].

Unexpectedly, the checkerboard artefacts from transpose convolutions, described in Section 2.3, did not noticeably degrade segmentation performance. Resize convolutions have become fairly prevalent among published methods to avoid this issue. However, our proposed method produced better segmentations when using transpose convolutions. Additionally, models with transpose convolutions trained ∼15% quicker as a result of containing ∼1.6 million fewer parameters.

Once the development phase was complete, our best model configuration was evaluated on a public dataset which has been used as a benchmark for several SOTA methods. The results published by Huang et al. [4], Zhang et al. [5], Gao et al. [6] and Gou et al. [7] are shown in Table 2 for comparison. From these comparison results on the MICCAI’15 set, we can see that our proposed model performed competitively with the SOTA models. The model of Gao et al. performs very well in the HD95 metric and was best for the brainstem, left and right parotid glands. Our method was best in the HD95 metric for the mandible. In the ASD/ mDTA metric our model performs best for all of the brainstem (0.81±0.31mm), mandible (0.20±0.08mm), left (0.77±0.14mm) and right parotid glands (0.81±0.28mm). The methods of Zhang et al. and Gao et al. share honours for the DSC score results, however, all five approaches perform closely. The external validation additionally confirmed that our model was more widely applicable than just the original model development dataset. Amjad et al. recently proposed a custom HN auto-segmentation CNN with a similar Res-UNet3D architecture to ours [28]. However, this model was trained with the MICCAI’15 dataset and 24 additional CT scans so we could not include their results in Table 2.

Our method has been specifically developed to leverage limited data, allowing for custom models to be trained on small datasets to segment different OARs or according to an updated protocol. Protocol-specific models can then be deployed in applications such as retrospective modelling studies or clinical trials to improve consistency. A natural extension for this study would be to further evaluate how model performance changes as the size of the training set changes. Siciarz et al. recently explored this question, showing segmentation performance to degrade as the number of training examples decreased [29]. However the fewest number of training samples considered by Siciarz et al. was still almost twice the size of the dataset used in this study.

In this study, we showed that through careful tuning and customisation a 3D CNN can be trained with a small dataset to segment the mandible, parotid glands and spinal cord with an accuracy that is similar to the magnitude of inter-clinician deviation. We evaluated our proposed model on a popular public dataset and produced high-quality segmentation results that were competitive with current state-of-the-art methods in multiple metrics.

## Declaration of Competing Interest

The authors declare that they have no known competing financial interests or personal relationships that could have appeared to influence the work reported in this paper.
